# Pharmacokinetics of piperaquine and its association with intermittent malaria preventive therapy outcomes during pregnancy

**DOI:** 10.1186/s40360-024-00762-6

**Published:** 2024-07-08

**Authors:** Eulambius M. Mlugu, Omary M.S. Minzi, Mats Johansson, Appolinary A. R. Kamuhabwa, Eleni Aklillu

**Affiliations:** 1 Department of Pharmaceutics and Pharmacy Practice, School of Pharmacy, Muhimbili University of Health and Allied Sciences, P. O, Box 65013, Dar es Salaam, Tanzania; 2 Department of Clinical Pharmacy and Pharmacology, School of Pharmacy, Muhimbili University of Health and Allied Sciences, P. O, Box 65013, Dar es Salaam, Tanzania; 3 Department of Laboratory Medicine, Karolinska Institutet at Karolinska University Hospital, Huddinge, Stockholm, 141 86 Sweden; 4 Department of Global Public Health, Karolinska Institutet, Karolinska University Hospital, Stockholm, Sweden

**Keywords:** Dihydroartemisinin-piperaquine, Malaria in pregnancy, Pharmacokinetics, IPTp

## Abstract

**Background:**

Dihydroartemisinin-piperaquine (DHP) recently showed superior effectiveness over sulfadoxine-pyrimethamine for malaria intermittent preventive treatment in pregnancy (IPTp). We investigated day 7 piperaquine pharmacokinetics and its therapeutic efficacy in preventing malaria during pregnancy.

**Methods:**

Malaria-free (mRDT) pregnant women (*n* = 400) who received monthly IPTp-DHP were enrolled and followed till delivery. Day 7 Plasma piperaquine concentrations were determined after each IPTp dose using UPLC/MS/MS. IPTp outcomes (symptomatic malaria and parasitemia during pregnancy, placental malaria, and maternal malaria at delivery) were monitored. Linear mixed model and Cox regression were used to assess predictors of day 7 piperaquine concentration and treatment outcome, respectively.

**Results:**

The incidences of symptomatic malaria and parasitemia during pregnancy per 100 person-year at risk were 2 and 33, respectively. The prevalence of histopathologically confirmed placental malaria and maternal malaria at delivery were 3% and 9.8%, respectively. Repeated monthly IPTp-DHP resulted in significantly increased day 7 plasma piperaquine concentration (*p* < 0.001). Following the 1st, 2nd, and 3rd monthly IPTp-DHP doses, the proportions of women with day 7 piperaquine concentration below the therapeutic threshold (< 30 ng/mL) were 6.1%, 4.1% and 3.6%, respectively. Factors such as maternal age, body weight and trimester were not significant predictors of day 7 piperaquine concentration. However, having a low day 7 piperaquine plasma concentration (< 30 ng/mL) was significantly associated with a higher risk of parasitemia during pregnancy (*p* = 0.004).

**Conclusion:**

Lower day 7 piperaquine plasma concentration is a risk factor for parasitemia during pregnancy. Single plasma sampling at day 7 can be used to monitor piperaquine effectiveness during IPTp-DHP.

**Trial registration:**

Registered 09/12/2016, PACTR201612001901313.

## Background

Malaria during pregnancy remains a major public health problem and a significant risk to maternal and neonatal mortality, maternal anemia, preterm birth, and low birth weight (LBW). In sub-Saharan Africa (SSA), more than 12 million pregnant women were exposed to malaria infection in 2022, and about 900,000 malaria-associated LBW [1]. Tanzania is among one of the 12 countries that accounted for 70% of the global estimated case burden and ranks at top among the 4 countries that collectively contribute to more than 50% of the global estimated deaths from malaria in 2022 [1]. Apart from interventions like insecticide-treated bed nets (ITNs) and indoor residual spraying, monthly intermittent preventive treatment in pregnancy with sulfadoxine-pyrimethamine (IPTp-SP) is recommended for all pregnant women during routine antenatal care (ANC) visits in malaria-endemic countries [2]. However, concerns persist regarding the effectiveness of IPTp-SP in providing malaria protection due to the rising resistance of *P*. *falciparum* against sulfadoxine-pyrimethamine (SP) across SSA, including Tanzania [3, 4].

Recent randomized clinical trials have demonstrated the superior effectiveness of dihydroartemisinin-piperaquine (DHP) over SP for intermittent preventive treatment during pregnancy (IPTp). In Kenya, a trial comparing IPTp-DHP given at an interval of 4–6 weeks versus the standard IPTp-SP reported that monthly IPTp-DHP was superior to IPTp-SP for prevention of placental malaria and parasitemia during pregnancy [5]. Similarly, two randomized clinical trials from Uganda reported the superiority of monthly IPTp-DHP compared to IPTp-SP for preventing placental malaria and parasitemia during pregnancy [6, 7]. In a randomized clinical trial conducted in an area with moderate malaria transmission in Tanzania, we recently reported the superiority of IPTp-DHP over IPTp-SP in preventing malaria during pregnancy and improving birth weight outcomes [8]. Despite the established effectiveness of monthly IPTp-DHP, this did not entirely prevent placental malaria and parasitemia during pregnancy. Lower piperaquine plasma exposure could likely be a potential risk factor for parasitemia in pregnant women receiving IPTp-DHP.

Physiologic changes in pregnancy alter the pharmacokinetic properties of many drugs including antimalarials. Some studies compared the pharmacokinetics of DHP administered for the treatment of uncomplicated *falciparum* malaria between pregnant and non-pregnant women [9–14]. While some studies found no significant difference in piperaquine exposure between pregnant and non-pregnant women [9, 10, 12, 14], one of the studies reported a similar total exposure to piperaquine, but a shorter terminal elimination half-life in pregnant women compared to non-pregnant women [9].

Very few studies have investigated the pharmacokinetics of piperaquine administered for malaria prevention during pregnancy. A previous study reported piperaquine target trough plasma concentrations of 10.3 ng/mL and 13.9 ng/mL to provide 95% and 99% protection respectively against *P. falciparum* during pregnancy [15]. In a recent study, it was found that 9.45% of pregnant women had trough piperaquine concentrations below the suggested target concentration (10.3 ng/mL) after three rounds of monthly IPTp-DHP [16]. Furthermore, another study reported a 72% higher piperaquine elimination clearance in pregnant women on IPTp as compared to post-partum women [17].

Changes in piperaquine plasma exposure during pregnancy may impact its effectiveness in preventing malaria. Monitoring the day 7 plasma concentration of the long-acting component of the antimalarial drug combination is recommended to assess treatment efficacy [18, 19]. Similar to lumefantrine in the artemether-lumefantrine drug combination, day 7 plasma concentration significantly correlates with total piperaquine exposure and area under the concentration curve (AUC), which is a crucial determinant of response to antimalarial drugs [20]. A day 7 plasma concentration of 30 ng/mL serves as a cutoff point for piperaquine, below which treatment failure is predicted [21]. However, further investigation is needed to determine whether day 7 plasma piperaquine concentration can predict the effectiveness of intermittent DHP therapy in preventing against malaria during pregnancy. Here, we have assessed the day 7 pharmacokinetics of piperaquine, its predictors, and its influence on IPTp outcomes, specifically parasitemia during pregnancy and at delivery, in pregnant women receiving DHP for malaria prevention in Tanzania.

## Methods

### Study design and population

This study was a prospective pharmacokinetics cohort nested within a two-arm Randomized Controlled Trial that compared monthly IPTp-DHP versus monthly IPTp-SP in Tanzania [8]. Pregnant women exposed to IPTp-DHP drug were enrolled and included in this pharmacokinetics study, and their blood samples were collected for pharmacokinetics analysis during the clinical trial. In brief, malaria-free pregnant women (mRDT) attending their first ANC were enrolled prospectively and received a full therapeutic dose of a once-daily fixed oral combination of DHP (D-ARTEPP, Batch S0160103, Guilin Pharmaceutical Co. Ltd, China) for three consecutive days. Each tablet contained 40 mg of dihydroartemisinin and 320 mg of piperaquine. The first dose was administered as directly observed therapy (DOT) at the ANC, while the second and third doses were taken at home 24th and 48th hours later, respectively. IPTp dose was administered at the monthly scheduled ANC visit until delivery. Self-reported adherence at the day 7 visit was recorded to assess adherence to the two doses administered at home.

### Monitoring treatment outcome

Finger prick blood samples were collected at enrollment and at every scheduled monthly visit for screening of malaria. Pregnant women were screened for malaria (by malaria Rapid Diagnostic Test [mRDT] and PCR) before enrollment and at each scheduled monthly visit. Blood samples were collected on Whatman filter paper (Whatman, Inc. NJ, USA), air-dried, and stored in a plastic bag at -80 ^o^C for screening of malaria parasite using PCR. During an unscheduled visit, malaria was diagnosed by mRDT and microscopy. All confirmed patent malaria cases during follow-up were treated with artemether-lumefantrine according to the national malaria guideline [22]. At delivery, malaria was screened from placental tissue using histopathology and from placental blood, maternal venous blood, and cord blood using mRDT, microscopy, and PCR. Birth weight and adverse birth outcomes were recorded immediately after birth. Congenital anomalies, neonatal and maternal death were monitored up to six weeks post-delivery.

### Chemicals and reagents

Piperaquine Tetraphosphate and Piperaquine-d6 Tetraphosphate were purchased from Toronto Research Chemicals (Toronto, ON, Canada). Acetonitrile (LC-MS grade) and methanol (LC-MS grade) were purchased from Fisher Scientific Co. (Beerse, Belgium). Triethyl ammonia (LC-MS grade) was purchased from Sigma*-*Aldrich (Missouri, USA). Formic acid (Optima™ LC-MS grade) was purchased from Fisher Scientific Co. (Brno-Černovice, Czech Republic). Healthy human plasma was obtained from the clinical Pharmacology laboratory at Karolinska University Hospital (Huddinge, Stockholm, Sweden).

### Quantification of plasma piperaquine concentration

Three ml of venous blood were collected on day 7 following administration of each IPTp-DHP dose for quantification of plasma piperaquine concentration. Plasma was prepared by centrifugation of whole blood at 2000 × g for 10 min, aliquoted, and stored at -80 ^o^C. Plasma samples, packed in dry ice, were shipped to the Department of Laboratory Medicine, Karolinska Institutet in Stockholm, Sweden, for measurement of plasma piperaquine concentration.

Plasma piperaquine concentration was determined using Ultra liquid chromatography-tandem mass spectrometry (UPLC-MS/MS) as previously described [23] with minor modifications. In brief, the stock solutions of piperaquine and internal standard (piperaquine-d6) were prepared in acetonitrile-water (1:9, v/v) containing 0.5% formic acid. Calibration standard samples (15.63, 62.5, 250, 1000, and 10,000 ng/mL) and quality control (QC) samples (31.25, 125, and 500 ng/mL) were prepared in blank plasma from two different stock solutions. A plasma sample (50 µL) and 50 µL of the internal standard piperaquine-d6 solution (100 ng/mL prepared in acetonitrile: water1:9 v/v and 0.5% formic acid) was added into 300 µL methanol, briefly vortex-mixed, and centrifuged at 25,000 g for 5 min. The supernatant (100 µL) was transferred to a 96-well plate placed on an autosampler, and 10 µL was injected into the UPLC-MS/MS for analysis.

Chromatographic separation was done on an ACQUITY BEH C18 column 2.1 × 50 mm, 1.8 μm (Waters, Milford, Massachusetts, USA). Elution was done using Mobile Phase A- 0.1% triethyl ammonia in ultrapure water and Mobile Phase B-0.1% triethyl ammonia in acetonitrile in a gradient mode at a flow rate of 0.6 mL/min. The analytes were eluted using a linear gradient, starting at 40% solvent B (0 min), isocratic hold (0–1 min), then increased from 40 to 90% solvent B (1–3 min), and then to 95% solvent B (3–3.1 min), hold (3.1–4.1 min), and then back to 40% solvent B (4.1–4.2 min). The total run time was 5 min, but the compounds were eluted after two minutes. Quality control samples (three samples, at a concentration of 31.25, 125, and 500 ng/mL) spiked in blank plasma were analyzed in triplicate within each batch of clinical samples to ensure the accuracy and precision of the assay. The coefficient of variation in precision and accuracy was below 10%. The lower limit of quantification (LLOQ) was 15.63 ng/mL. The calibration curve was fitted with least square linear regression weighted by 1/x with a correlation coefficient (r) = 0.99. The method was validated according to the European Medicines Agency Guideline on bioanalytical method validation [24].

### Data analysis

Treatment outcome variables were presented as prevalence (n, %) and incidence per person-year at risk. Piperaquine plasma concentration was log10 transformed before statistical analysis using parametric tests. Comparisons of log_10_ Piperaquine plasma concentration after receiving the 1st, 2nd, and 3rd IPTp doses were done using a paired t-test. The Cox regression model and Kaplan Meir plot with log Rank test were used to explore independent predictors of parasitemia during pregnancy. A Linear Mixed Model was used to examine independent predictors of log10 day 7 piperaquine concentration. Univariate followed by multivariate logistic regressions were used to explore factors associated with any parasitemia at delivery and adverse birth outcomes. Variables with *p*-value ≤ 0.2 in the univariate analysis were included in the multivariate model. Statistical analyses were performed using IBM SPSS Statistics for Windows, Version 27.0 (Armonk, NY: IBM Corp). Graph Pad Prism version 8.3 for Windows (Graph Pad, La Jolla, CA, USA) was used for graphical presentations. P values < 0.05 were statistically significant.

### Ethical approval

The study received ethical approval from the Institutional Review Boards of the Tanzania National Institute for Medical Research (Ref. No. NIMR/HQ/R.8a/Vol.IX/2342), the Muhimbili University of Health and Allied Sciences (Ref. No. 2016-06-07/AEC/Vol.XI /2), and Stockholm Ethics Committee (Ref. No. 2020 − 00857). Written informed consent was obtained from all study participants before enrollment.

## Results

### Study participants’ characteristics and IPTp outcome

The baseline participants’ demographic characteristics are listed in Table 1. The primary study outcome (histopathological placental malaria at delivery) was collected from 400 pregnant women. A total of 245 pregnant women who were part of a recent clinical trial [8] provided 505 plasma samples for piperaquine pharmacokinetics analysis.

**Table 1:** Baseline characteristics of pregnant women (*n* = 400)

Variables		Frequency
**Age category** (n, %)	Adolescent (< 20 years)	75 (18.7)
	Young adult (20–34 years)	253 (63.3)
	Adult (> 35 years)	72 (18.0)
**Gravidity at enrollment** (n, %)	Primigravida	92 (23.0)
	Secundigravida	90 (22.5)
	Multigravida	218 (54.5)
**Gestational age at enrollment (weeks)** (n, %)	Early (13–20 weeks)	175 (43.8)
	Late (≥ 21 weeks)	225 (56.2)
**Trimesters at enrollment** (n, %)	Second (13–27 weeks)	370 (92.5)
	Third (≥ 28 weeks)	30 (7.5)
Median age (Interquartile range) Years		26 (20–32)
Median gestational age (Interquartile range) weeks		21 (20–24)
Median weight (Interquartile range) kg		55 (50–60)

The incidence of symptomatic malaria and parasitemia during pregnancy, and the prevalence of parasitemia, placental malaria and adverse birth outcomes at delivery are presented in Table 2.

**Table 2:** Malaria and adverse birth outcomes during pregnancy and at delivery (*n* = 400)

Outcomes	Frequency
**Parasitemia during pregnancy**	
Symptomatic malaria during pregnancy^**+**^ (95% CI)	2 (0.3 to 6)
Parasitemia during ANC^**+**^ (95% CI)	33 (21 to 41)
**Parasitemia at delivery**	
Histopathological placental malaria (active & past) % (n)	3.0 (12)
Histopathological placental malaria [parasites] (active only) %, (n)	1.5 (6)
Histopathological placental malaria [pigments] (past only) %, (n)	1.5 (6)
*Any parasitemia at delivery % (n)	9.8 (39)
Any adverse birth outcomes % (n)	22.5 (90)

^**+**^Incidence per 100 person-year at risk; *Any malaria at delivery detected from placental tissue by histopathology, from a maternal, cord, or placental blood by RDT, microscopy, or PCR; CI = 95% Confidence interval

### Day 7 piperaquine plasma concentration

**Fig. 1:**
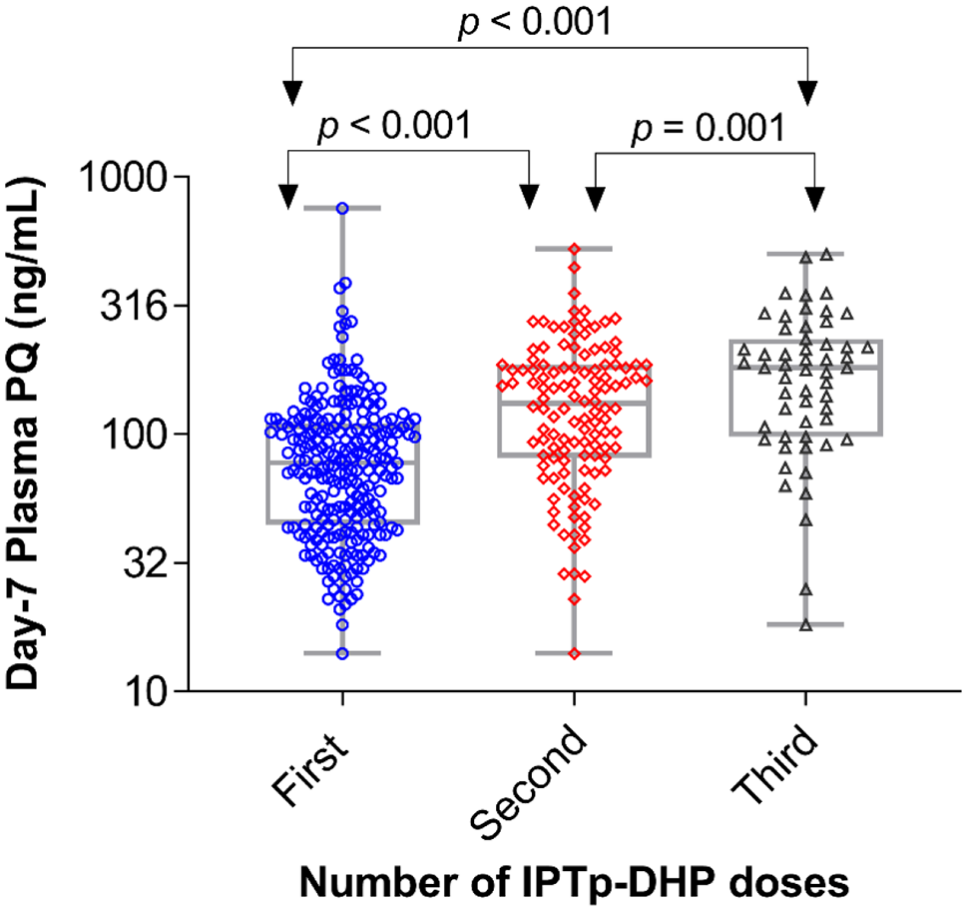
Comparison of geometric means of day-7 plasma piperaquine concertation after the first, second and third IPTp-DHP doses using paired t-test. The box plots show the means ± SD, while whiskers denote the minimum and maximum values

There was wide inter-individual variation in day 7 plasma piperaquine concentration ranging from 15 ng/mL to 759 ng/mL. The median day 7 piperaquine concentration with interquartile range after the first IPTp-DHP dose was 77 (45 to 110) ng/mL. After the second and third IPTp-DHP doses, the median day 7 concentrations with interquartile range were 132 (82 to 187) ng/mL and 180 (98 to 236) ng/mL, respectively. Repeated monthly IPTp-DHP dose resulted in significantly increased day-7 plasma piperaquine concentration over time (*p* < 0.001). A comparison of the geometric mean of day 7 plasma piperaquine concentration between the first, second and third IPTp doses is presented in Fig. 1.

After the 1st IPTp dose, 15/245 (6.1%, 95% CI = 3.7 to 9.9) women had piperaquine day 7 concentration < 30 ng/ml, the target concentration to predict piperaquine therapeutic efficacy. After the second and third monthly IPTp-DHP doses, 5/122 (4.1%, 95% CI = 1.8 to 9.2) and 2/55 (3.6%, 95% CI = 1 to 12) women had piperaquine day 7 concentration < 30 ng/mL, respectively.

### Predictors of day 7 piperaquine concentration over time

The overall predictors of Log10 day 7 piperaquine concentration were explored by including socio-demographic and clinical characteristics. Log10 day 7 piperaquine concentrations after the first and second IPTp-DHP doses were included in the model as dependent factors. Maternal age, body weight, gravidity and trimester were not significant predictors of log day 7 piperaquine concentration in the univariate and multivariate analysis (Table 3).

**Table 3:** Linear mixed model estimates for predictors of log day 7 piperaquine concentration

Variable	Log day 7 concentration			
	Univariate analysis		Multivariate analysis	
	Estimate (95% CI)	*p*-value	Estimate (95% CI)	*p*-value
Weight (kg)	-0.001 (-0.003 to 0.01)	0.75		
**Age (years)**				
Adult (≥ 20)	1 (Reference)	0.42		
Adolescent (< 20)	-0.03 (-0.10 to 0.02)			
**Gravidity**				
Multigravida	1 (Reference)	0.15	1 (Reference)	0.23
Primigravida	-0.05 (-0.12 to 0.02)		-0.04 (-0.12 to 0.03)	
**Trimester**				
Third	1 (Reference)	0.06	1 (Reference)	0.06
Second	-0.11 (-0.22 to 0.004)		-0.10 (-0.21 to 0.01)	

CI 95% confidence interval

### Predictors of parasitemia during pregnancy

The influence of baseline characteristics and day 7 piperaquine concentration on the risk of malaria during pregnancy was evaluated. Maternal age, gravidity, gestational age and body weight were not found to be significantly associated with the risk of parasitemia during pregnancy (Table 4). On the other hand, women with a day 7 piperaquine concentration < 30 ng/ml after the first IPTp-DHP dose had a 5 times higher risk of parasitemia during pregnancy compared to those with a concentration ≥ 30 ng/mL, in both the univariate (*p* = 0.004) and multivariate analysis (*p* = 0.003). Similarly, women with piperaquine day 7 concentration < 30 ng/mL after the second IPTp-DHP dose had a 4.4 times higher risk for parasitemia during pregnancy compared to women with ≥ 30 ng/mL at the baseline although not significantly (*p* = 0.05) (Table 4). In the multivariate model, day 7 piperaquine concentration after the first and second IPTp-DHP doses were included differently to avoid collinearity.

**Table 4:** Cox-regression results for predictors of parasitemia during pregnancy

Variable	Malaria during pregnancy				
	Univariate analysis			Multivariate analysis	
	Cumulative risk *n*/*N* (%)	Hazard Ratio (95% CI)	*p*-value	aHR (95% CI)	*p*-value
Age (years)	33/400 (8.3)	0.99 (0.97 to 1.04)	0.88		
Gravidity	33/400 (8.3)	0.82 (0.66 to 1.01)	0.06	0.81 (0.60 to 1.09)	0.17
Gestational age (weeks)	33/400 (8.3)	0.93 (0.83 to 1.03)	0.16		
Weight (kg)	33/400 (8.3)	0.99 (0.97 to 1.03)	0.67		
**Day 7 PQ cutoff after the first IPTp dose**					
< 30 ng/mL	4/15 (26.7)	5.22 (1.70 to 16)	**0.004**	5.60 (1.81 to 17.33)	**0.003**
≥ 30 ng/mL	13/230 (5.7)	1 (Reference)		1 (Reference)	
**Day 7 PQ cutoff after the second IPTp dose**					
< 30 ng/mL	2/5 (40)	4.40 (0.94 to 20)	**0.05**	4.40 (0.94 to 20.53)	**0.05**
≥ 30 ng/mL	9/117 (7.7)	1 (Reference)		1 (Reference)	

95% CI = 95% confidence interval; bolded *p* values indicate significant association; aHR = adjusted hazard ratio

**Fig. 2:**
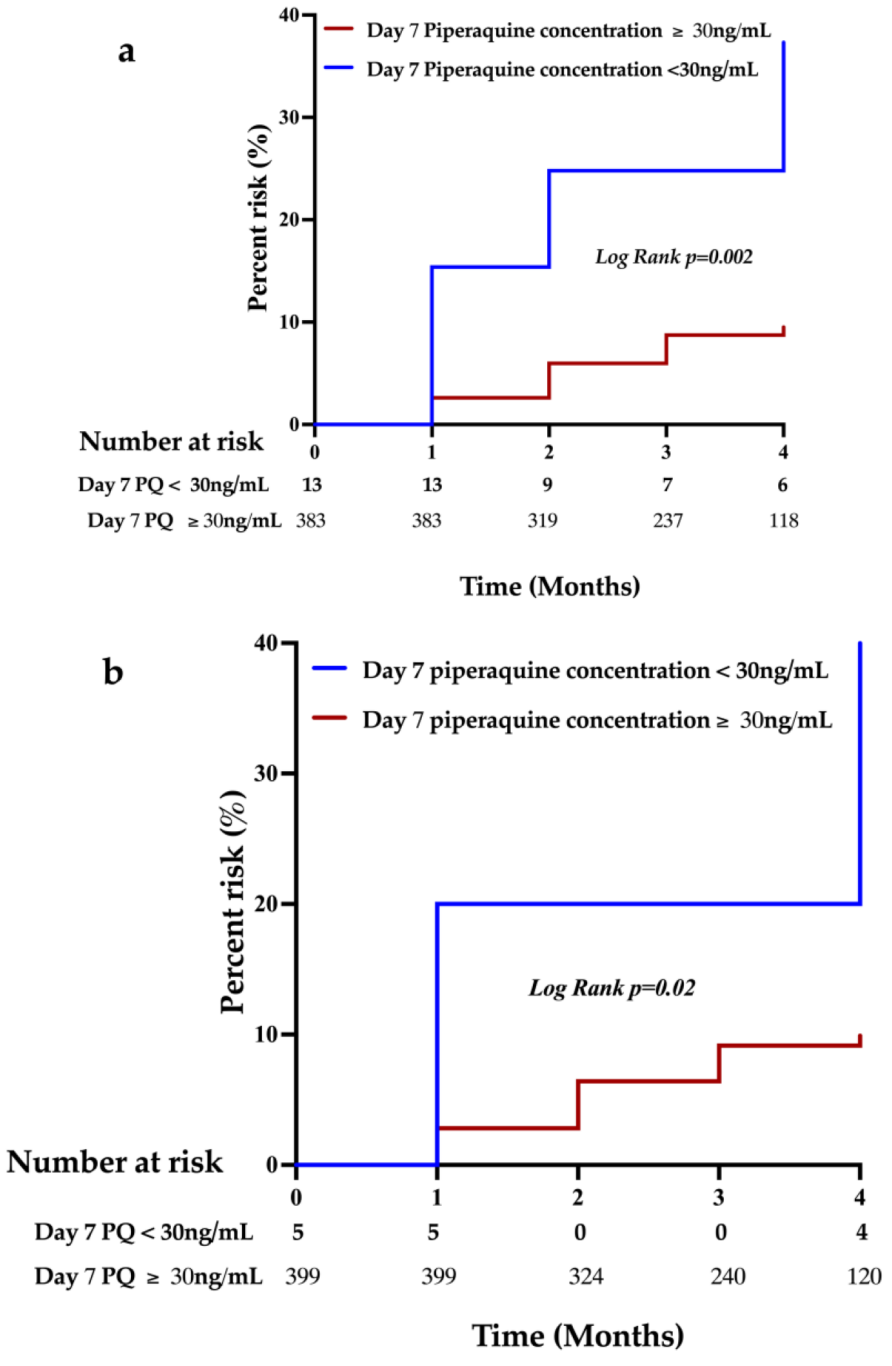
Hazard ratios for parasitemia positivity during pregnancy stratified by day 7 piperaquine concentration < 30 ng/mL versus ≥ 30 ng/mL after the first IPTp-DHP dose (**a**) and after the second IPTp-DHP dose (**b**)

Kaplan Meir plot indicated a significantly higher risk of parasitemia over time during pregnancy in women with day 7 piperaquine concentration < 30 ng/ml both after the first and the second IPTp-DHP doses (log-rank *p* < 0.05) as compared to women with ≥ 30 ng/mL (Fig. 2).

Maternal age, gravidity and gestational age were not significantly associated with any parasitemia at delivery or adverse birth outcome both in univariate and multivariate logistic regression analysis.

## Discussion

This prospective cohort study investigated the day 7 pharmacokinetics of piperaquine and its association with the effectiveness of IPTp-DHP in preventing malaria during pregnancy. Study participants received a full therapeutic dose of DHP monthly until delivery and were regularly monitored for malaria during pregnancy and at delivery. Our key findings include (i) a significant association of lower day 7 plasma piperaquine concentration (< 30 ng/mL) with a higher risk of parasitemia during pregnancy (ii) a significant increase in day 7 piperaquine plasma concentration with advancing gestational age during pregnancy over time (iii) a high proportion of pregnant women attained piperaquine plasma concentration above the lower therapeutic threshold (30 ng/mL). To the best of our knowledge, this is the first study to explore the pharmacokinetics of day 7-piperaquine during IPTp-DHP and its impact on malaria prevention during pregnancy.

Our result indicates a significant association between lower day 7 piperaquine concentration (< 30 ng/mL) with a higher risk of parasitemia during pregnancy. Day 7 piperaquine concentration of 30 ng/mL has been established as a threshold concentration to predict *P. falciparum* treatment efficacy [19]. Piperaquine concentration below 30 ng/mL at day 7 has been associated with treatment failure in *P. falciparum*-infected malaria patients [21, 25, 26]. The result of our study suggests that day 7 piperaquine plasma concentration can also predict the effectiveness of DHP in preventing malaria in pregnancy. Indeed, we found a significant association between day 7 piperaquine concentration < 30 ng/mL and a higher risk of parasitemia (failure in preventing malaria) during pregnancy (Table 4). This finding suggests that the threshold day 7 piperaquine concentration of 30 ng/mL could be utilized to monitor the effectiveness of IPTp-DHP. Our finding implies that sufficient piperaquine plasma concentration at day 7 is crucial to eliminate any sub-patent parasitemia and prevent new infections. Therefore, day 7 piperaquine concentrations < 30 ng/mL may not prevent malaria infection occurring in the three weeks before the next IPTp-DHP.

In this study, we found a high proportion of pregnant women achieved day 7 piperaquine concentration of ≥ 30 ng/mL after the first, second and third monthly IPTp-DHP doses. Despite the differences in study designs, the finding of this study is comparable to the previous study which reported that 90.6% of women who received monthly IPTp-DHP in Kenya and Indonesia achieved piperaquine trough concentration above the pre-established target concentration (10.3 ng/mL) [16], sufficient to provide 95% malaria protection [15]. The higher proportion of women with adequate day 7 piperaquine concentration (≥ 30 ng/mL) during IPTp-DHP suggests that monthly IPTp with a three-day treatment course of DHP is optimal for ensuring adequate protection against malaria during pregnancy. Although some women (< 7%) had day 7 piperaquine concentrations below < 30 ng/mL, this does not necessarily justify adjusting the DHP dose at the first IPTp to maintain the target concentration. This is because increasing the DHP dose at the first IPTp could substantially increase piperaquine concentration throughout IPTp-DHP duration, potentially leading to safety concerns such as QTc prolongation. Other determinants of piperaquine bioavailability such as genetic variation and adherence to monthly IPTp-DHP require further investigation to be made.

Our linear mixed model revealed that the trimester did not significantly predict day 7 piperaquine pharmacokinetics. Piperaquine is primarily metabolized by both CYP3A and CYP2C8 enzymes [27]. Recently, we reported a significant increase in CYP3A enzyme activity from the second to the third trimester, indicating higher drug metabolism in the third trimester compared to the second trimester [28]. The lack of trimester impact on piperaquine day-7 pharmacokinetics may be due to the involvement of metabolic pathways other than CYP3A, and possibly the repeated administration of DHP. This finding is in line with previous studies that found no significant impact of trimester on the pharmacokinetics of piperaquine [9, 16]. Overall, these results suggest that a consistent DHP dose regimen could be given throughout IPTp duration regardless of gestational age at the first ANC. Furthermore, we could not find a significant association between body weight and day 7 piperaquine pharmacokinetics, consistent with a previous finding [29], suggesting that weight-based DHP dosing may not be necessary during IPTp. However, future studies should consider body weight based on existing knowledge and previously published findings [14].

In this study, we did not include non-pregnant women; thus, we could not assess the influence of pregnancy on day 7 piperaquine concentration. A previous study reported significantly higher day 7 piperaquine concentration in pregnant women compared to non-pregnant women, although no significant difference was observed in overall piperaquine exposure [14]. Similarly, another study in Sudan reported significantly higher piperaquine exposure in pregnant women after the first dose as compared to non-pregnant women, with no significant difference in total piperaquine exposure between the two groups [12]. In a recent IPTp study in Uganda, pregnant women were found to have lower piperaquine exposure (72% higher piperaquine clearance) compared to postpartum women [17]. Similar findings were reported in Papua New Guinea, indicating 45% higher clearance of piperaquine in pregnant women compared to non-pregnant women, with no significant difference in the overall plasma piperaquine exposure [9]. The reported increased clearance of piperaquine could be attributed to the increased CYP3A enzyme activity during pregnancy, as previously reported [28].

This study has some limitations. Firstly, only pregnant women in the second and third trimesters were included. This is because IPTp is recommended to begin at least in the second trimester [30]. Therefore, the power to detect the effect of trimester on day 7 piperaquine plasma concentration might be limited, since pregnant women in the first trimester may have different physiological characteristics affecting drug disposition compared to those in the second and third trimesters. Additionally, the overall piperaquine exposure was not analyzed since a single time point sampling on day 7 was collected. However, several studies indicated that day 7 piperaquine plasma concentration suitably predicts antimalarial therapeutic efficacy and correlates well with AUC.

## Conclusions

We report a significant association between lower day 7 piperaquine plasma concentrations and a higher risk of parasitemia during pregnancy. Furthermore, day 7 piperaquine plasma concentration significantly increases with the increasing number of repeated IPTp-DHP doses. A large proportion of women attained the target day 7 piperaquine concentration (≥ 30 ng/mL), indicating that monthly IPTp-DHP is effective for optimal malaria protection during pregnancy.

## Data Availability

The datasets used and/or analyzed during the current study are available from the corresponding author on reasonable request.

## Abbreviations

ANC: Antenatal Care
AUC: Area Under the Concentration time Curve
CYP: Cytochrome P450 enzymes
DHP: Dihydroartemisinin-piperaquine
DoT: Direct observed Therapy
ITN: Insecticide Treated bed Net
LBW: Low Birth Weight
mRDT: Malaria Rapid Diagnostic Test
MUHAS: Muhimbili University of Health and Allied Sciences
NIMR: National Institute for Medical Research
PCR: Polymerase Chain Reaction
SP: Sulfadoxine-pyrimethamine
SSA: Sub-Sahara Africa
UPLC-MS/MS: Ultra liquid chromatography-tandem mass spectrometry
WHO: World Health Organization
IPTp: Intermittent Preventive Treatment in pregnancy
